# Comprehensive Review of Non-Operative Management of Hallux Rigidus

**DOI:** 10.7759/cureus.987

**Published:** 2017-01-20

**Authors:** Charles Kon Kam King, James Loh SY, Qishi Zheng, Kinjal V Mehta

**Affiliations:** 1 Orthopaedics, Changi General Hospital; 2 Department of Epidemiology, Singapore Clinical Research Institute

**Keywords:** hallux rigidus, osteoarthritis, management, non-operative, intra-articular injection, manipulative therapy, physiotherapy, orthosis

## Abstract

This article aims to provide an evidence-based literature review for the non-operative management of hallux rigidus. Currently, there is very little article on the evidence for the non-operative management of hallux rigidus. A comprehensive evidence-based literature review of the PubMed database conducted in November 2016, identified 11 relevant articles out of 560 articles assessing the efficacy of non-operative modalities for hallux rigidus. The 11 studies were then assigned to a level of evidence (I-IV). Individual studies were reviewed to provide a grade of recommendation (A-C, I) according to the Wright classification in support of or against the non-operative modality. Based on the results of this evidence-based review, there is poor evidence (grade C) to support use of intra-articular injections for pain relief for a period of three months and fair evidence (grade B) against the use of intra-articular injections for long term efficacy. There is poor evidence (grade C) to support manipulation and physical therapy and poor evidence (grade C) to support modifications in footwear, insoles and orthotics. There were no good evidence (grade A) recommending any interventions. In general, most of the interventions showed improvement. However, the evidence is poor in recommending orthosis, manipulation and intra-articular injections. There is a need for high-quality Level I randomized controlled trials with validated outcome measures to allow for stronger recommendations to be made. There is no study that looked solely at the use of pharmaceutical oral agents for the treatment of hallux rigidus. Non-operative management should still be offered, prior to surgical management.

## Introduction and background

Hallux rigidus was first described in 1887 by Daves-Colley [1]. Hallux rigidus has an estimated incidence of one in 40 in patients aged over 50 years [2]. The first ray is an important weight-bearing part of the foot. During the normal stance phase of the gait cycle, the hallux bears twice the load compared with the lesser toes and approximately 40% to 60% of the body weight [3]. Forces on the first ray are increased during sporting activity with approximately two to three times the body weight during running and up to eight times body weight in running jumps [4].

Excessive length of the first ray increases the stress concentrated at the metatarsophalangeal (MP) joint during toe-off. People with a long first ray are more prone to developing hallux rigidus [5]. Many other etiologies have been postulated including trauma, abnormally elevated first metatarsal and a positive family history; however, most cases are likely idiopathic [6].

The symptoms of hallux rigidus include swelling, joint pain, and stiffness associated with restricted dorsiflexion. At the beginning, the joint swelling is due to synovitis. With the progression of the condition, osteophytes develop around the joint margins, specifically on the dorsal aspect. This further restricts joint motion.

Plain radiographs are used to grade the severity of hallux rigidus. Hattrup and Johnson’s classification and Coughlin and Shurnas’s classification are the most commonly used ones [7-8]. In grade one of Hattrup and Johnson’s classification, there are mild changes with a maintained joint space and minimal spurring. In grade two, there are moderate changes, joint space narrowing, the bony proliferation of the metatarsal head, and phalanx and subchondral sclerosis or cysts. In grade three, there are severe changes with moderate to severe joint space narrowing, extensive bony proliferation, and loose bodies or a dorsal ossicle.

The management of hallux rigidus initially is non-operative. In those that have failed non-operative treatment, surgical treatment would be offered. Foot orthoses and shoe modification such as limiting the bending of the toe box with the full-length rocker can limit dorsal irritation. In addition, other modalities such as physiotherapy, manipulative therapy, and nonsteroidal anti-inflammatory drugs as well as intra-articular injections form part of our current armamentarium for the non-operative management of hallux rigidus. Surgical management of hallux rigidus would include cheilectomy, arthrodesis, arthroscopy, osteotomy and total, partial or resection arthroplasty.

The aim of this review was to evaluate the different non-operative treatment modalities for hallux rigidus thereby providing a clinical guideline based on the available scientific evidence. There is a little review of the evidence for the non-operative management of hallux rigidus.

## Review

### Sources of information and search strategy

A comprehensive literature review was conducted using the PubMed database. We followed the guidelines proposed by PRISMA declaration (Preferred reporting items for systematic reviews and meta-analyses) [9].

Search interval: up to 1st November 2016.

Selection of studies

Search criteria in non-operative modality with the following keywords in English: “Hallux rigidus”, “non-operative treatment”, “physiotherapy”, “injection”, “orthotic”, “chiropractic therapy” and “conservative management”.

Shortlisted articles were reviewed to identify studies with non-operative treatment and examining their results.

Exclusion criteria were

Case series with less than five cases were also excluded. Non-English publications were excluded.

Final selection criteria

**Figure 1:**
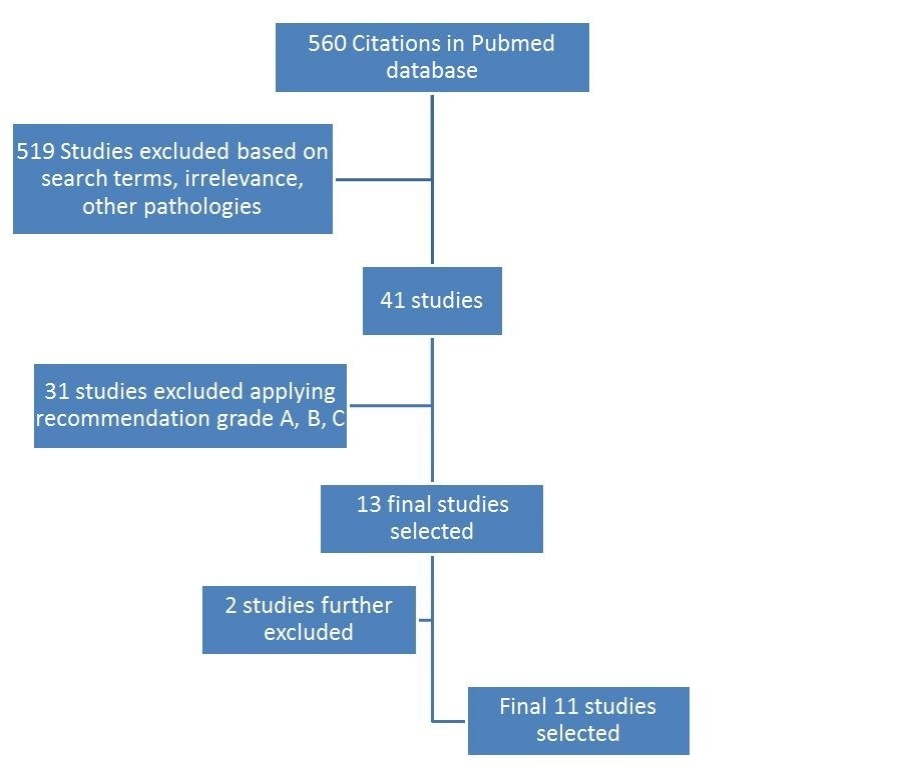
PRISMA flow diagram of non-operative modalities

For inclusion in this study, each study was then assigned a level of evidence (I-V) in accordance with the standards of the Journal of Bone and Joint Surgery [10]. The literature was reviewed by two foot and ankle surgeons and a grade of recommendation (A-C, I) were assigned to each intervention based on the classification of Wright [11]. (Tables 1-2) Studies with recommendation grade A, B, and C were reviewed (Figure 1).

**Table 1:** Levels of evidence for non-operative studies

Level	Therapeutic studies investigating results of treatment
I	High-quality randomized trials with statistically significant difference or no statistical difference but narrow confidence intervals; systematic reviews of level I randomized controlled trials (and study results were homogeneous)
II	Lesser quality randomized controlled trials (eg < 80% follow-up, no blinding, or improper randomization); prospective comparative studies; systematic review of level II studies or level I studies with inconsistent results
III	Case-control series; retrospective comparative studies; systematic reviews of level III studies
IV	Case series
V	Expert opinion

**Table 2:** Grades of recommendation for summaries or reviews of orthopaedic surgical studies

Grade	Description
A	Good evidence (level-I studies with consistent findings) for or against recommending intervention.
B	Fair evidence (level-II or III studies with consistent findings) for or against recommending intervention.
C	Conflicting or poor-quality evidence (level-IV or V studies) not allowing a recommendation for or against intervention.
I	There is insufficient or conflicting evidence not allowing a recommendation for or against recommending intervention.

Statistical analysis

A meta-analysis adopting random effect model was performed to synthesize the pre-post changes of VAS for both rest and walking pain, among studies evaluating hyaluronic acid injection [12-13]. Standardized mean difference (SMD) with 95% confidence interval (95%CI) was reported. Statistical heterogeneity was assessed by I² value. For I² value, 30% to 50% represented moderate heterogeneity, 50% to 80% substantial heterogeneity and > 80% considerable heterogeneity [14].

A qualitative synthesis was performed to evaluate various non-surgical interventions reported in the included studies.

The meta-analysis was conducted in Comprehensive Meta-analysis (Version 3.3, Biostat, Englewood, NJ).

### Results

The search returned 560 articles on hallux rigidus. There were 41 articles on non-operative modalities. After applying our exclusion criteria and applying the recommendation grade, there were 13 studies with recommendation grade A, B and C. Two studies were excluded as one study was retracted from the journal and one Cochrane review on this subject matter reviewed, one study that was already included in our shortlist. A total of 11 articles were assessed. There were only three articles that had a recommendation grade B or above.

The grade of recommendation assigned to each intervention is summarized in Table 3. Summary of the study characteristics and outcomes are presented in Table 4.

**Table 3:** Summary of grade of recommendation for or against non-operative modality for hallux rigidus

Intervention	Number of studies	Level I	Level II	Level III	Level IV	Grade	Recommendation
Modifications in footwear, insoles and orthotics	3	-	-	-	3	C	Poor quality of evidence recommending intervention
Manipulation and physical therapy	2	-	1	-	1	C	Poor quality of evidence recommending intervention
Joint injections (study for intervention)	6	-	-	-	3	C	Poor quality of evidence recommending intervention
Joint injections (study against intervention)		1	1	-	1	B	Fair evidence against intervention

**Table 4:** Summary of study characteristics and outcomes Abbrevations: VAS, visual analog score; M, months; Y, year; MPJ, metatarsophalangeal joint; GPS, Global Patient Satisfaction scale, AOFAS, American Orthopaedic Foot and Ankle Society score for hallux evaluation

Study (Level)	Design	Number	Follow up	Age (year, SD) or (year, range)	Gender (Female)	BMI	Intervention	Outcome	Treatment modality (Recommendation)
Grady JF, 2002 (IV) [15]	Case series	772	> 1 year	46 (17-78)	375 (49%)	NA	Conservative treatment (orthoses, corticosteroid, change in shoe)	428 (55%) responded, within which: 362 (84%) treated successfully with orthoses 42 (10%) with a change in shoes 24 (6%) with steroid injections. 296 (38%) required surgery 48 (6%) did not respond to conservative or surgery	Modifications in footwear, insoles and orthotics (Treatment provided pain relief)
Smith RW, 2000 (IV) [16]	Case Series	22	14.4 years	53 (25-71)	9 (39%)	NA	Self-care methods (most used a shoe with ample toe box)	63% would support original decision of non-operative treatment 16 feet (67%) had a measurable loss of cartilage space 92% of cases the pain level remained constant	Modifications in footwear, insoles and orthotics (Treatment provided did not worsen condition over time)
Welsh BJ, 2010 (IV) [17]	Single-arm trial	35	24 weeks	42.2, 11.5	26	24.4, 3.8	Foot orthoses	Foot function index (0-100): Change from baseline to 24 weeks: 14.5mm (P<0.001) Kinematic analysis: No systematic change in 1st MTP joint dorsiflexion or ankle/subtalar complex pronation	Modifications in footwear, insoles and orthotics (Treatment provided pain relief comparable to adequate analgesic response to treatment)
Shamus J, 2004 (II) [18]	Non-randomized controlled trial	20	4 weeks	32.8, 5.85	15 (75%)	NA	Sesamoid joint mobilization, flexor hallucis strengthening and gait training vs Various	MPJ passive range of motion: Control 14.4˚ ± 8.0˚ Intervention 42.7˚ ± 7.8˚ Flexor hallucis strength difference (Kg): Control 0.7 ± 0.4 Intervention 3.5 ± 1.0 Change of VAS for rest pain (0-10cm): Control: 2.6 ± 1.1 Intervention: 6.4 ± 1.3	Manipulation and physical therapy (Treatment provided pain relief)
Solan MC, 2001 (IV) [19]	Case Series	35	> 12 months	52.3, 11.04	NA	NA	Manipulation+ Bupivacaine	Symptomatic relief: Grade 1: median 6 months, 1/3 will require surgery Grade 2: median 3 months, 2/3 will require surgery Grade 3: Little or no symptomatic relief, surgical treatment planned within 3 months	Manipulation and physical therapy (Treatment provided pain relief)
Munteanu SE, 2011 (II) [21]	Randomized controlled trial	151	6 months	54.5, 11.3	56 (37%)	27.1, 3.8	Hylan G-F 20 vs Saline	Foot Health Status Questionnaire (FHSQ) HGF: (n=75) Baseline: 56.2, 19.3 1M: 67.5, 20.8 3M: 68.2, 22.5 6M: 68.0, 21.4 Placebo: (n=76) Baseline: 57.0, 17.8 1M: 69.7, 19.6 3M: 72.5, 17.0 6M: 71.4, 18.7	Intra-articular injection (Treatment did not provide pain relief. Treatment is similar to placebo)
Pons M, 2007 (I) [22]	Randomized controlled trial	37	12 months	62 (40 – 80)	31 (84%)	NA	Sodium hyaluronate (SH) (n=20) vs Triamcinolone acetonide (TA) (n=20)	VAS for rest pain (0-100mm): SH: Baseline: 62.2 ± 10.7 3M: 26.2 ± 23.9 TA: Baseline: 58.7 ± 11.6 3M: 34.1 ± 16.6 VAS for walking pain (0-100mm): SH: Baseline: 61.4 ± 13.0 3M: 24.2 24.1 TA: Baseline: 59.3 ± 12.2 3M: 36.8 ± 19.7 AOFAS SH: Baseline:51 3M:77.6 TA Baseline:48.2 3M:64.3	Intra-articular injection (Treatment provided pain relief at 3 months, however at 1 year SH 7/15 (46.6% required surgery and TA 9/17 (52.9%) require surgery)
Grice J, 2016 (IV) [23]	Case Series	365	24 months	41 (14 – 82)	NA	NA	Corticosteroid injection	Significant improvement (n=365) Overall 314 (86%) >3M: 202 (55%) >6M: 145 (39%) >2Y: 107 (29%) Significant improvement (n=22 for hallux rigidus) Overall 20 (91%) >3M: 3 (14%) >6M: 3 (14%) >2Y: 2 (9%) Required surgery: 88 (24%)	Intra-articular injection (Treatment provided did not offer pain relief for longer than 3 months)
Maher A, 2007 (IV) [24]	Case Series	16	12 months	NA	NA	NA	Hyaluronic acid injection	VAS for rest pain (0-10cm): Baseline: 6.2 (1-9) Post: 2.8 (0-8.5) Sig. improvement <1M: 3 (23%) <6M: 3 (23%) >6M: 6 (46%)	Intra-articular injection (Treatment provided pain relief)
Petrella RJ and Cogliano A, 2004 (IV) [25]	Single-arm trial	47	> 16 weeks	71, 4.3	0 (0%)	26.3, 1.7	Hyaluronic acid injection	VAS for rest pain (0-100mm): Baseline: 41.2 ± 3.1 4M: 30.4 ± 2.9 GPS: Baseline 3.1 4M: 4.51 VAS for walking pain (0-100mm): Baseline: 68.9 ± 5.9, 4M: 32.8 ± 3.1	Intra-articular injection (Treatment provided pain relief)
Steinberg MD, 1971 (IV) [26]	Case Series	100	NA	NA	NA	NA	Lidocaine injection	Response rate: 89 (89%) responded to the treatment	Intra-articular injection (Treatment provided pain relief)

Modifications in footwear, insoles and orthotics

Levels III-IV. The highest quality study examining the outcome of use of orthoses was a level III study (Table 4). Grady, et al. [15] reported that out of 772 patients with symptomatic hallux limitus (n=772), 428 (55%) patients were successfully treated conservatively with 362 (84%) of these 428 patients treated with the use of orthoses (level IV). Two hundred and ninty six (38%) patients required surgery and 48 (6%) patients did not respond to conservative management.

Smith, Katchis and Ayson  [16] conducted a longitudinal questionnaire-based study of 22 patients with 24 feet (n=24) with an average follow-up of 14.4 years (level IV). Thirteen patients managed with modifications to their shoes by using shoes with ample room in the toe box. Seven patients found that they had relief of their symptoms by avoiding high heels. Of all the patients, 63% would support their original decision of non-operative treatment. It was also found that pain level remained constant in 92% of cases over an average of 14.4 years and there was no correlation between subjective complaints and radiographic evidence of progression of hallux rigidus.

Welsh, et al. [17] conducted an observational study with a group treated with foot orthoses (n=35) with a follow-up of 24 weeks (level IV). The pain score as measured on the modified pain subscale of the foot function index (FFI) was 48 mm at baseline and improved to 14.5 mm at the end of 24 weeks (p <0001). The authors concluded that orthotic design could offer a reduction in mechanically induced pain to a level that is considered an adequate analgesic response to treatment. The author's study was supported in party through an unrestricted grant from the foot orthoses company.

Grade of recommendation. Based on the previously mentioned literature, the non-operative modality of modifications in footwear, insoles and orthotics is assigned a grade C recommendation (poor evidence, level IV or V studies with consistent findings) in treatment of hallux rigidus.

Manipulation and physical therapy

Levels II-IV. There was one level II study and one level IV study looking at manipulation and physical therapy as a treatment modality. Both studies were for the use of manipulation and physical therapy for the treatment of hallux rigidus. Shamus, et al. [18] conducted a prospective randomized study of an intervention group with physical therapy and manipulation (n=10) and a control group (n=10) over four weeks with a total of 12 therapy sessions (level II). The outcome measured was 1st metatarsophalangeal (MTP) joint range of motion which was 42.7˚ ± 7.8˚ in the intervention group compared to 14.4˚ ± 8.0˚ in the control group (p <0.001). Flexor hallucis strength difference from pretest and post-test of 3.5 Kg ± 1.0 in the intervention group and 0.7 Kg ± 0.4 in the control group (p <0.001). Lastly, the pain level difference based on a verbal analog scale of zero to 10 showed a difference of 6.4 ± 1.3 in the intervention group compared to 2.6 ± 1.1 in the control group (p <0.001). The authors concluded that for individuals aged between 26 to 43 years of age, this approach resulted in significant increase in range of motion, strength and function. There was also no adverse outcome reported.

Solan, Calder and Bendall [19] conducted a retrospective case series of 29 consecutive patients with 35 MTP joints (n=35) with manipulation under anaesthetic and injection of 40 mg of Depo-medrone made up in 3 ml of 0.5% bupivacaine with a minimum follow-up of one year (level IV). The authors subdivided the patients according to Karasick and Wapner radiographic classification [20]. They reported that patient with grade one had symptomatic pain relief for a median of six months, although 1/3 will require surgery. Those with grade two had pain relief for a median of three months with 2/3 requiring surgery and finally those with grade three had little to no pain relief with surgical treatment planned within three months.

Grade of recommendation. Based on the previously mentioned literature, the non-operative modality of manipulation and physical therapy is assigned a grade C recommendation (poor evidence, level IV or V studies with consistent findings) supporting it as an effective treatment for hallux rigidus.

Pharmaceutical therapy with joint injections

Levels II-IV. There were six studies that looked at intra-articular injections. Munteanu, et al. [21] conducted a randomized placebo controlled study on a group of patients given intra-articular hyaluronan (n=75) and placebo, saline (n=76) over six months (level II). The authors found that the pain score as measured from the foot pain domain of the Foot Health Status Questionniare (FHSQ) was 68.0 at six months for the intervention group and 71.4 at six months for the placebo group (p=0.312). They concluded that single intra-articular injection of hyaluronan was no more effective than placebo. In addition, they did not find any major safety issues with the injections. This study was funded by the Australian Podiatry Education and Research Foundation and La Trobe University Faculty of Health Sciences as well as the provider of the hyaluronan product.

Pons, et al. [22] conducted a single blind randomized study with a group treated with sodium hyaluronate injection (n=20) and another group treated with triamcinolone acetonide (n=20) over 12 months (level I). The authors treated patients who had grade one osteoarthritic change on radiograph according to the Karasick and Wapner classification. The treatment group with sodium hyaluronate had a mean visual analog score from baseline to end of study of 62.2 mm ± 10.7 to 26.2 mm ± 23.9 while the triamcinolone acetonide group had a visual analog score from baseline to end of study 58.7 mm ± 11.6 to 34.1 mm ± 16.6 (p <0.05). They concluded that both intra-articular injections resulted in decrease in pain and improvement of function at three months after injection. However, they found that at one year follow-up, there was a high percentage in both groups that required surgery. There were no major safety issues.

Grice, et al. [23] in their retrospective case series of a group treated with corticosteroid (0.5% Marcaine with 40 mg Depo-medrone) in the foot and ankle (n=365) over a period of two years (level IV). In the subgroup of patients with hallux rigidus (n=22) they found that 91% had pain relief but that the benefit lasting more than six months was just 14%. They concluded that corticosteroid injections appeared ineffective in providing significant pain relief for more than three months in conditions such as hallux rigidus.

Maher and Price [24] in their retrospective case series with a group treated with intra-articular sodium hyaluronate (n=16) over a period of one year (level IV) found that pain measured on the visual analog scale improved from a mean of 6.2 cm (range 1-9) pre-injection to 2.8 cm (range 0-8.5) post injection. Six out of 14 patients reported pain relief of at least six months since final injection. The authors concluded that intra-articular injection did appear to offer pain relief but did mention that the small number in study subjects. No adverse effect was reported.

Petrella and Cogliano [25] conducted a prospective study with a group, with the treatment with the intra-articular injection of hyaluronic acid (n=47) with a minimum follow of 16 weeks (level IV). They reported pain relief from baseline to follow-up at 16 weeks from 41.2 mm ± 431 to 30.4 mm ± 2.9 (p <0.01). They concluded that hyaluronic acid did provide pain relief.

Steinberg [26] conducted a retrospective case series of a group treated with intra-articular weekly injections of lidocaine (n=100, level IV). He concluded that 11 patients did not respond to the treatment and went on to have surgery. The period of follow-up and the outcome measures were not reported.

Grade of recommendation. Based on the previously mentioned literature, the non-operative modality of pharmaceutical therapy including joint injections is assigned a grade B recommendation (fair evidence, level II or III studies with consistent findings) against it as an effective treatment for hallux rigidus. There is one study (level II) that show that intra-articular injections are no more effective that placebo and another study (level I) that shows that it is effective at three months, but by one year, nearly half of the patients would require surgery. In addition, there is one study (level IV) that shows that injections are not effective for pain relief after three months. However, there are three studies (level IV) that show that injections do work for treatment of hallux rigidus.

Meta-analysis on hyaluronic acid injection

**Figure 2:**
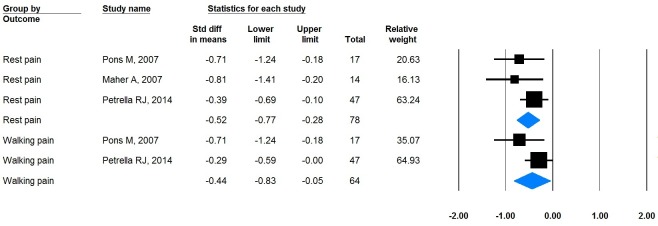
Forest plot of VAS for both rest pain and walking pain

Three studies reported VAS of rest pain before and after the injection and three reported VAS of walking pain. Significant reliefs from the injection were observed in both VAS measures, in which pooled VAS of rest pain was -0.52 (95%CI: -0.77, -0.28) and walking pain was -0.32 (95%CI: -0.52, -0.11) (Figure 2).

Both I2 of VAS of rest pain and walking pain were zero percent and 10.2%, suggesting that the included studies were in low heterogeneous.

### Discussion

We have reviewed the non-operative options for hallux rigidus and evaluated the level of evidence based on the most current available literature. Due to the limited studies available, we performed a qualitative summary of the current evidence of non-surgical interventions.

Overall, most of the interventions showed improvement after the intervention. It was shown that a combination of sesamoid joint mobilization, flexor hallucis strengthening and gait training lead to a significant increase in flexor hallucis range of motion, strength and function compared to traditional treatment. The systematic review by Brantingham, et al. [27] reported a grade C recommendation for manipulative therapy of the ankle and/or foot combined with multimodal or exercise therapy for plantar fasciitis, metatarsalgia, and hallux limitus/rigidus. This further confirms the evidence for this treatment modality.

Hyaluronic acid injection, as synthesized by the meta-analysis, showed a significant improvement on both rest and walking pain level. Six patients (46%) in Maher A, 2007 studies reported a significant improvement lasting more than six month. Steinberg MD, 1971 reported an 89% response rate after receiving injection with 2 cc of 2% lidocaine (Xylocaine) at weekly intervals. However, there are two studies (level I and level II) that showed that injections are not effective on the long term over one year and one study (level IV) that showed that its pain relief was only effective up to three months.

There were no standardized endpoints across included studies, six studies reported visual analogue scale (VAS) and other continuous index for measuring the pain level, two studies reported kinematic analysis on the 1st MTP joint motion, and five studies reported various binary endpoint (Yes/No) on pain relief within a certain period. There was only one non-operative modality (good evidence, level one study).  The majority of evidence available was based on level IV studies. None of the studies looked into the sole use of oral analgesics as a treatment of hallux rigidus.

A potential source of bias is the inclusion of studies with different radiological grades of hallux rigidus. This can lead to a biased inclusion and selection. Additionally, longer follow-up periods and larger study groups would provide better evidence.

## Conclusions

Based on the results of this review, there is fair evidence (grade B) against the use of the non-operative treatment of intra-articular injections. There is poor evidence (grade C) in support of modifications of footwear, insoles and orthotics and poor evidence (grade C) in support of manipulation and physical therapy.
